# Mechanic-Driven Biodegradable Polyglycolic Acid/Silk Fibroin Nanofibrous Scaffolds Containing Deferoxamine Accelerate Diabetic Wound Healing

**DOI:** 10.3390/pharmaceutics14030601

**Published:** 2022-03-10

**Authors:** Shenfang Zha, Yohanes Kristo Sugiarto Utomo, Li Yang, Guizhao Liang, Wanqian Liu

**Affiliations:** Key Laboratory of Biorheological Science and Technology, Ministry of Education, College of Bioengineering, Chongqing University, Chongqing 400030, China; shenfang202202@163.com (S.Z.); k188831347756@163.com (Y.K.S.U.); yangli_cqu@163.com (L.Y.)

**Keywords:** mechanical property, extracellular matrix, diabetic wound healing, biodegradable, secondary damage

## Abstract

The extracellular matrix (ECM), comprising of hundreds of proteins, mainly collagen, provides physical, mechanical support for various cells and guides cell behavior as an interactive scaffold. However, deposition of ECM, especially collagen content, is seriously impaired in diabetic wounds, which cause inferior mechanical properties of the wound and further delay chronic wound healing. Thus, it is critical to develop ECM/collagen alternatives to remodel the mechanical properties of diabetic wounds and thus accelerate diabetic wound healing. Here, we firstly prepared mechanic-driven biodegradable PGA/SF nanofibrous scaffolds containing DFO for diabetic wound healing. In our study, the results in vitro showed that the PGA/SF-DFO scaffolds had porous three-dimensional nanofibrous structures, excellent mechanical properties, biodegradability, and biocompatibility, which would provide beneficial microenvironments for cell adhesion, growth, and migration as an ECM/collagen alternative. Furthermore, the data in vivo showed PGA/SF-DFO scaffolds can adhere well to the wound and have excellent biodegradability, which is helpful to avoid secondary damage by omitting the removal process of scaffolds. The finite element analysis results showed that the application of silk fibroin-based scaffolds could significantly reduce the maximum stress around the wound. Besides, PGA/SF-DFO scaffolds induced collagen deposition, re-vascularization, recovered impaired mechanical properties up to about 70%, and ultimately accelerated diabetic wound healing within 14 days. Thus, our work provides a promising therapeutic strategy for clinically chronic wound healing.

## 1. Introduction

Diabetes mellitus (DM) affects approximately 422 million patients worldwide [1]. About 25% of diabetic patients will eventually develop a diabetic foot ulcer (DFU) [1,2]. DFU, which ultimately leads to limb loss, disability, and even death, has caused serious medical, economic, and social burdens worldwide [3]. Normal skin wound healing is an intrinsic fundamental physiological process for ensuring the integrity of the skin and contains several overlapping stages: hemostasis, inflammation, proliferation, and remodeling [4]. In the proliferative phase, the extracellular matrix (ECM), as an interactive scaffold, provides physical, mechanical support for adhesion, proliferation, and migration of various cells and thus promotes tissue regeneration in the wound sites, including angiogenesis [5,6,7]. During normal wound healing, ECM remodeling, involving a balance between formation, degradation, and maturation, is vital for optimum wound healing [8,9]. However, accumulating evidence has proved that the highly orchestrated processes in diabetic wound healing are badly impaired [10], which leads to a reduction in ECM deposition, especially collagen content, inferior mechanical properties of the diabetic wound, and ultimately delayed chronic wound healing [11,12,13]. Therefore, it is critical to develop ECM/collagen alternatives to remodel the mechanical properties of the diabetic wound and thus accelerate diabetic wound healing.

Electrospinning technology has a long history and its first report dates back to the 18th century. The development of nanotechnology promoted the prosperity of the electro-static spinning technique in tissue engineering [14]. Electrospun nanofibrous scaffolds are of special importance in tissue engineering and have gained massive attention in the past years owing to their biomimetic native ECM structure, high surface area, porous three-dimensional nanofibrous structures, etc. [15]. Furthermore, electrospun nanofibrous scaffolds containing drugs, cytokines, and cells have been already reported to promote diabetic wound healing. For example, Subramani Kandhasamy et al. fabricated the PHB-GEL-OSA-COL nanofibrous scaffold to mimic extracellular matrix and enhance wound healing [16]. In addition to mimicking the ECM structure, Guo and colleagues confirmed that α-Lactalbumin-based nanofiber dressings improved burn wound healing and reduced scarring through promoting proliferation and adhesion of fibroblasts and collagen I synthesis, and decreasing α-SMA expression [17]. Moreover, another desirable therapeutic target for wound healing is to induce angiogenesis. Chen et al. reported deferoxamine (DFO)-loaded electrospun composite fibrous membranes (chitosan (CS) and polyvinyl alcohol (PVA)) rapidly accelerated diabetic wound healing by recruiting angiogenesis relative cells [18]. Thus, electrospun nanofibrous scaffolds are considered one of the most promising biomaterials for diabetic wound healing. However, conventional nanofibrous scaffolds still have some disadvantages and limit their applications, such as poor mechanical properties [19], high immunogenicity [20], poor stability [21], and slow rate of degradation [22]. Among them, one of the most important shortcomings is that some wound dressings need to be removed [15,23], which easily causes secondary damage before the wound is completely healed. This is because exudates or cells in wound beds already adhere to the wound dressing, making it difficult to remove the wound dressings during long-term contact [24]. Additionally, some materials degrade slowly and occupy wound space for a long time, which hampers the formation of a new blood vessel network [18]. Therefore, the development of new, biocompatible, and biodegradable biomaterials is of great practical significance to relieve pain and further accelerate diabetic wound healing. 

Silk fibroin (SF), an FDA-approved well-known natural polymer, is derived from the most common silkworm, *Bombyx mori* (*B. mori*). SF poses many unique characteristics, mainly including superior mechanical property [12], excellent spinnability, high biocompatibility, biodegradability, hydrophilic property, environmental stability, etc. [25,26,27]. Hence, SF has been widely applied in the biomedical and pharmaceutical fields in the forms of SF-based suture material of surgery [28], electrospun nanofibrous scaffolds [3], hydrogels [29], etc. Although pure SF-based scaffolds have already been proved to be appropriate for skin repair, combining SF with other biomaterials would endow it more potential for diabetic wound healing [30]. Polyglycolic acid (PGA) is the raw material of poly (glycolic acid) sutures and consists of glycolic acid, produced by the process of metabolism in humans. PGA is broadly applied in the biomedical fields owing to its promising properties such as hydrophilicity, biocompatibility, biodegradability, and nontoxicity [31]. Hence, blending of both PGA and SF would endow the as-prepared scaffolds with suitable biodegradability to avoid secondary damage and leave enough space for the formation of a new blood vessel network with wound healing. 

Weak vascularization is considered as a key factor for impaired diabetic wound healing [32]. In the past decades, many treatment strategies have already attempted to improve vascularization in diabetic wound healing [29]. Among them, DFO, an FDA-approved iron chelator for iron overload diseases [33], and an angiogenic agent [34], has attracted researchers’ attention. DFO can facilitate vascularization by upregulating hypoxia-inducible factor-1α (HIF-1α) and further inducing the expressions of other downstream angiogenic factors, such as vascular endothelial growth factor (VEGF) [35] and stromal cell-derived factor 1 (SDF-1α) [29]. However, accumulating evidence reveals that DFO has a short plasma half-life, thus requiring frequent infusion of DFO for a long time to achieve the expected effect [36]. Unfortunately, high doses of DFO easily lead to severe side-effects due to its toxicity [37,38]. Therefore, it would be desirable to develop a drug delivery system for sustained-release of DFO during diabetic wound healing.

Against this background, we hypothesized that nanofibrous scaffolds, consisting of PGA, SF, the raw materials of sutures, and DFO, an angiogenic agent, could not only potentially remodel the mechanical properties of the diabetic wound to provide mechanical support for cells and further guide cell behavior as a temporary and transitional ECM/collagen alternative, but also release DFO carried in the scaffolds to induce angiogenesis and further accelerate diabetic wound healing. Meanwhile, nanofibrous scaffolds will be moderately biodegradable due to the addition of PGA, which will successfully avoid secondary damage by omitting the process of scaffolds removal and make more space for re-vascularization. Based on the hypothesis above, we designed and prepared mechanic-driven biodegradable PGA/SF nanofibrous scaffolds containing DFO (PGA/SF-DFO) and further investigated the characteristics and wound repair effect of as-prepared nanofibrous scaffolds in vitro and in vivo.

## 2. Materials and Methods

### 2.1. Cell Isolation and Culture

Primary human umbilical vein endothelial cells (HUVECs) were isolated from human umbilical cord veins according to a method by the author of [39] with slight modifications. Briefly, the cells were firstly isolated by 10% collagenase I and then cultured in M199 medium (HyClone, South Logan, UT, USA) containing 20% fetal bovine serum (FBS, Biological Industries, Kibbutz Beit Haemek, Israel), 3.2 ng/mL β-ECGF (Sigma, Billerica, MA, USA), 0.108 mg/mL heparin sodium (Solarbio, Tongzhou, Beijing, China), 2.5 ng/mL thymidine (Solarbio, Beijing, China), and 100 U/mL penicillin/streptomycin. For all cell experiments, early passages (passages 2~7) were used. All cells were maintained at 37 °C in an atmosphere of 95% O_2_/5% CO_2_.

### 2.2. Fabrication of PGA/SF and PGA/SF-DFO Nanofibrous Scaffolds 

PGA, was purchased from Sigma-Aldrich (St. Louis, MO, USA). Rough SF without silk sericin was obtained from cocoons *B. mori* (Chongqing Sericulture Science and Technology Research Institute, Chongqing, China) according to a method previously described with some modifications [26]. Briefly, cocoons *B. mori* were firstly cut into pieces and boiled in water containing 0.2 M Na_2_CO_3_ for 1 h and then washed three times with distilled water. Subsequently, rough silk fibroin was dissolved into 9.3 M lithium bromide (LiBr) (Sigma-Aldrich, Shanghai, China) and stirred for 4 h at 55 °C. After stirring, a dialysis bag (MW = 3500 Da, Pierce, Rockford, IL, USA) was used to purify the mixture. Finally, SF was freeze-dried and then saved in a vacuum. 

PGA/SF nanofibrous scaffolds were fabricated by electrostatic spinning using the 10% (*w*/*v*) PGA/SF mixture solution according to our previous study with slight modifications [40]. Briefly, 0.25 g of PGA and 0.25 g SF were dissolved in 5 mL hexafluoroisopropanol (HFIP) and then the mixture was stirred for three days at room temperature. Next, PGA/SF nanofibrous scaffolds were prepared by the electrospinning device. In brief, the solution was firstly loaded in a 10 mL plastic syringe with a 25-G blunt stainless steel needle (Small Parts, Inc., Logansport, IN, USA). The syringe was then put on the device. PGA/SF nanofibrous scaffolds were obtained by a high voltage source (Dongwen High Voltage Power Supply Plant, Tianjin, China), which supplied 15 kV of high voltage. At the same time, flow speed was controlled at 1 mL/h using a syringe pump (Longer Precision Pump, Baoding, China). Finally, the nanofibrous scaffolds were collected by the aluminum foil with a size of 15 cm (width) × 38 cm (length), which was covered on a collector drum. The speed of the roller was controlled at 250 r/min. After electrospinning, the nanofibrous scaffolds were dried in a vacuum for later use. 

PGA/SF-DFO scaffolds were obtained by 1-Ethyl-3-(3-(dimethylamino)propyl) carbodiimide HCl (EDC, Aladdin, Shanghai, China)/N-hydroxysuccinimide (NHS, Aladdin, Shanghai, China) method [41,42]. Briefly, PGA/SF scaffolds were firstly cut into discs with different diameters. After that, aluminum foil was required to tear out, and then the membranes were put into a clear dish to prepare PGA/SF-DFO scaffolds. DFO was cross-linked into the scaffolds in the (2-(N-morpholino) ethanesulfonic acid) hydrate (MES) buffer (0.1 M, pH = 6, Solarbio, Beijing, China) for 24 h by adding the EDC/NHS mixture. After that, the PGA/SF-DFO nanofibrous scaffolds were washed with distilled water three times. Finally, all samples were frozen at −20 °C overnight and freeze-dried for 48 h.

### 2.3. Physicochemical Characterization of PGA/SF and PGA/SF-DFO Nanofibrous Scaffolds

#### 2.3.1. Surface Morphology Analysis

The morphology of all scaffolds was studied by using a field emission scanning electron microscope (Nova 400 NanoSEM FEI, Philips, The Netherlands). Before detection, the samples were firstly put on a metal holder through the black conducting resin, and then coated gold for 30 s. Next, all samples were scanned by SEM using an accelerating voltage of 10 kV. The diameters of images were analyzed with Image J software (National Institutes of Health, Bethesda, MD, USA).

#### 2.3.2. Hydrophilicity Analysis

To analyze the hydrophilicity of scaffolds, the device named MagicDroplet Model200 (Taiwan, China) was chosen to detect static water contact angles (WCA) of scaffolds. Briefly, distilled water was firstly loaded in the vertical plastic syringe and then dripped 5 μL onto the dry samples. After dripping water for about 5 s, images were quickly captured, and then WCA was obtained. 

#### 2.3.3. Chemical Component Analysis

Fourier transform infrared (FTIR) spectroscopy was employed for observing special characteristic peaks in the PGA/SF and PGA/SF-DFO nanofibrous scaffolds. Briefly, the samples were subjected to 32 scans at a 4 cm^−1^ resolution over the wavenumber range of 500 to 4000 cm^−1^. The peaks were obtained by a Nicolet spectrometer system (System 2000, Perkin-Elmer, Waltham, MA, USA). At the same time, X-ray photoelectron spectroscopy (XPS) was used to analyze the element component and variation at the scaffold by Avantage software (Version 5.952, Thermo Fisher Scientific, UK). 

#### 2.3.4. Mechanical Test In Vitro

To evaluate the mechanical properties of scaffolds, an electromechanical dynamic/static testing system (Instron E1000, High Wycombe, UK) was chosen to measure the macro-tensile with pneumatic grips as a previous study described, with slight modifications [40]. Briefly, all the scaffolds were firstly folded into a rectangular strip with a size of 2.5 cm (length) × 0.4–0.5 cm (width) × 0.03–0.04 cm (thickness) and subsequently loaded in the machine. The thickness of the scaffolds was obtained by using an electronic micrometer. The parameters of measurement were set as follows: the force loaded and crosshead speeds were 50 N and 10 mm/min, respectively. Simultaneously, the initial grip separation was set at 1 cm. All the data including ultimate tensile strength and characteristic parameters such as Young’s modulus, stress-strain curves, and strain at break, were obtained by the Bluehill^®^ 2 software and then processed by using the Origin 8.0 software. 

#### 2.3.5. Drug Release In Vitro

The release of DFO from composite scaffolds was accomplished in vitro according to a previous study with slight changes [42]. Briefly, 10 mM DFO was chosen to chemically cross-link or physically absorb into PGA/SF nanofibrous scaffolds. Then nanofibrous scaffolds were immersed in 1 mL PBS (pH 7.4) on a shaker at 37 °C for five consecutive days with a speed of 100 rpm. Next, the total PBS in each well was collected and refilled at each specific time interval. Finally, the concentration of DFO in the collected samples was measured by the reaction between DFO and ferric chloride (FeCl_3_) (Sigma-Aldrich, Shanghai, China), and the results were obtained by using a UV spectrophotometer at 485 nm.

#### 2.3.6. Scaffolds Degradation In Vitro 

In order to estimate the degradable properties of the scaffolds in vitro, PGA/SF and PGA/SF-DFO scaffolds were immersed in 20 mL PBS (pH 7.4) on the shaking incubator at 37 °C at a speed of 100 rpm for two weeks. At 0, 3, 7, 10, and 14 d, the scaffolds were washed with distilled water three times and dried at 37 °C overnight. After that, the mass of scaffolds was weighed. To further examine the morphology analysis of scaffolds in the degradation process, the degraded scaffolds were observed by SEM at 0 and 7 d, as described in Section 2.3.1.

### 2.4. Effects of PGA/SF and PGA/SF-DFO Scaffolds on HUVECs In Vitro

#### 2.4.1. HUVECs Adhesion In Vitro 

5 × 10^3^ cells/well of HUVECs were seeded in a 24-well plate for analyzing the adhesion capacity of HUVECs on the scaffolds. After 24 h, the scaffolds were taken out to wash with PBS three times, followed by fixing in 4% paraformaldehyde (Solarbio, Beijing, China) for 30 min at room temperature. The paraformaldehyde was removed and rinsed with PBS three times again. Subsequently, the scaffolds were stained with 4′,6-diamidino-2-phenylindole (DAPI) (Beyotime, Shanghai, China), a usual nuclear dye for 15 min at room temperature. After that, all samples were washed and then sealed with an anti-fluorescence quenching agent. Ultimately, the results were obtained microscopically with a Live Cell Imaging System (Leica DMI 6000B, Leica, Germany). In our displayed data, the adhered HUVECs were counted by Image-Pro Plus 6.0 software (Media Cybernetics, Rockville, MD, USA).

#### 2.4.2. Cell Viability and Proliferation

To explore the cytotoxicity of all the scaffolds on the viability and proliferation of HUVECs, an MTS assay using a CellTiter 96 AQueous One Solution Cell Proliferation Assay (MTS, Promega, Madison, WI, USA) was carried out in triplicate according to the manufacturer’s instructions. In this assay, the density of 3 × 10^3^ cells per well of HUVECs were seeded onto the fabricated scaffolds and then cultured in a 96-well plate for 1, 2, and 3 days. At the end of incubation, the medium was removed and replaced with fresh media containing 20% MTS without FBS. After incubating for 3 h in the dark, 100 μL of the mixed supernatant was transferred to another new well and OD value was measured at 490 nm. The results were collected and processed by applying the following formula: OD=ODt−OD0, where ODt represents the OD value of the experimental group and OD0 represents the OD value of blank wells. For each assay, six parallel wells were set. The displayed data is the mean value of three independent experiments.

#### 2.4.3. Migration Assay In Vitro

In order to determine the effect of PGA/SF and PGA/SF-DFO scaffolds on the migration of HUVECs, the transwell chamber migration assay was performed in vitro. Briefly, 700 μL medium containing 5% FBS was added to each well, which contained sterile scaffolds. Then, 300 μL serum-free medium containing 1 × 10^4^ HUVECs/well were seeded in the transwell chamber with membrane pore diameter of 8 μm (Corning, NY, USA). After culturing for 24 h, the upper chamber was slightly taken out to remove the inserts and then washed three times with PBS. Next, the non-migrated cells on the upper surface of the chamber were wiped out with a cotton-tipped applicator. After that, the migrated cells on the bottom of the chamber were carefully fixed in 4% paraformaldehyde for 30 min at room temperature. Finally, all samples were stained with 0.01% crystal violet (Solarbio, Beijing, China) for 15 min at room temperature. The images were captured by a light microscope and counted with Image-Pro Plus 6.0 software.

#### 2.4.4. Tube Formation Assay In Vitro

DFO can promote tube formation in vitro as previously reported, so PGA/SF- DFO scaffolds were thought to have the ability to induce the tube formation. To confirm the effect of scaffolds on tube formation of HUVECs, the tube-formation assay in vitro was processed. In brief, 200 μL of ice-cold Matrigel matrix (Corning, NY, USA) was firstly added to the well in a 24-well plate and covered evenly at 4 °C for 30 min. Then the matrix was solidified at 37 °C for 30 min, followed by HUVECs were harvested, and then re-suspended in serum-free medium at the final concentration of 2 × 10^4^ cells/mL. Subsequently, 250 μL suspension was transferred to the gel and cultured at 37 °C for 12 h. Eventually, the images about tube formation were collected by a light microscope and the total length of tube formation was processed by Image-Pro Plus 6.0 software. 

### 2.5. Wound Healing Evaluation In Vivo

#### 2.5.1. Induction of Diabetic Mice

Diabetic mice were induced according to previous studies with some modifications [43,44,45,46]. Briefly, male ICR mice (body weight: 28–35 g, eight-week-old) were obtained from HUNAN SJA LABORATORY ANIMAL CO., LTD. All mice were maintained on a standard laboratory diet and water ad libitum. All protocols about animal experiments were approved by the Ethics Committee of Chongqing University Three Gorges Hospital (protocol code 202122 and date of approval 4 March 2021) and carried out according to the National Institutes of Health Guidelines for the Care and Use of Laboratory Animals (NIH publication number 80–23, revised 1996). All the mice were observed for one week before diabetes induction and then the initial blood glucose (BG) level was measured after fasting for a night. The mice were divided into two groups: the normal and diabetic groups, respectively. To generate a diabetic model, the mice were fed high-fat food for four weeks, while those mice in the control group were fed a normal diet. After that, streptozotocin (STZ; 35 mg/kg/day, sigma) dissolving in the citrate buffer (pH 4.2–4.5) or citrate buffer alone was intraperitoneally injected for five consecutive days. On the 10th day after injection, the glucose level was checked by using a glucometer (Roche Diagnostics GmbH, Mannheim, Germany). Only mice with glucose level above 300 mg/dL were identified as diabetic [47]. 

#### 2.5.2. Excisional Wound Model Preparation 

The excisional wound model was established based on a previous method [48]. Before the operation, the mice were anesthetized by intraperitoneal injection of 10% chloral hydrate solution (Sinopharm Chemical Reagent Co., Ltd., Shanghai, China). Subsequently, a punch biopsy (6 mm diameter) was selected to create the wound on their dorsal scapular region. 

#### 2.5.3. Scaffolds Degradation In Vivo

The scaffolds degradation in the diabetic wound was conducted to observe the biodegradability of scaffolds in vivo. In brief, the fluorescein isothiocyanate (FITC, Sigma) labeled scaffolds were fabricated by physically absorbing or cross-linking using the EDC/NHS method, as described in Section 2.2. Then the nanofibrous scaffolds were covered on the wound bed in a dark environment. At 3 and 5 d, the samples were harvested and embedded in the Optimal Cutting Temperature (OCT) compound followed by cryo-sectioned (Thermo, Cryotome FSE, MA, USA) at 8 μm. The images were obtained by a confocal microscope (LSM 780, Carl Zeiss, Jena, Germany). 

#### 2.5.4. Wound Closure Measurement

The diabetic mice were observed for another four weeks and then randomly divided into three groups: control, PGA/SF, and PGA/SF-DFO group, separately. The wounds were covered with sterilized scaffolds where the control group was treated with nothing. The images of wound closure were recorded on days 0, 7, and 14 d after surgery. The wound area at every indicated interval was analyzed using Image J software. The percentage of wound closure was calculated by using the following formula: $\mathrm{Wound}\;\mathrm{closure}\;\left(\%\right)=\left[\left(A0-\mathrm{At}\right)/A0\right]*100$, where A0 shows the initial wound area at day 0 and At represents the wound area at time t, such as 7 and 14 d, respectively.

#### 2.5.5. Finite Element Modeling Analysis

Finite element modeling analysis of wound tissue was performed using ABAQUS (standard version 16.4; Dassault Systèmes SIMULIA Corp., Johnston, RI, USA). In brief, the geometry of the skin model was determined on histologic specimens and previous literature [49,50,51,52,53]. Skin was modeled as a combination of the (1) epidermis and dermis layer (800 kPa), (2) subcutaneous layer (100 kPa), and (3) silk fibroin (5 MPa). The Poisson ratio was set at 0.49 for all layers. An initial 6 mm diameter wound model was created at day 0 and the diameters of wound models at day 3 and 5 were determined by different wound closure rates. In addition, meshing or discretizing all the elements was carried out with eight-node linear hexahedron elements (C3D8R by ABAQUS/Standard). Silk fibroin sub-models with different thicknesses were established to simulate the degradation of scaffolds over time. Subsequently, the whole model was created to calculate the local distribution of stress. Detailed analysis of maximum stress value was carried out by extracting the part of epidermis and dermis layer from the global model using the sub-model technique of ABAQUS.

#### 2.5.6. Histologic Analysis 

Hematoxylin-eosin (H&E) and Masson’s trichrome staining were carried out on day 7 after operation to further estimate the effect of scaffolds on diabetic wound healing in vivo. All samples were taken from the wound sites and fixed in 4% (*v*/*v*) paraformaldehyde solution at 4 °C overnight and embedded in paraffin. All prepared samples were cut at 5 µm for subsequent staining as a common method. All stained samples were captured by using a light microscope. Besides, immunohistochemical staining and immunofluorescence (IF) staining were carried out with CD31 (Abcam, Great Britain; 1:200) or CD31 +α-smooth actin (α-SMA) (Abcam, Great Britain; 1:50) to observe tube formation in vivo, respectively. 

#### 2.5.7. Mechanical Property Evaluation In Vivo

A mechanical test in vivo was carried out to further evaluate the repair effect of scaffolds on diabetic wound healing. Briefly, unwounded normal, diabetic skin and the wound bed on day 14 were cut into a rectangular strip with a size of 2.5 cm (length) × 0.4 cm (width) × 0.03–0.035 cm (skin thickness). Then the mechanical property of fresh skin was measured as described in Section 2.3.4 with slight modifications. The ends of fresh skin were fixed between filter paper using adhesive tape to prevent slipping.

### 2.6. Statistical Analysis 

The mean ± standard error (SEM) was used to display all data. Data analysis was completed by GraphPad version v8.01 software. Student′s *t*-test and ANOVA were employed to evaluate the statistical significance and *p*-value (<0.05) was considered statistically significant.

## 3. Results and Discussion

### 3.1. Fabrication and Characterization of PGA/SF-DFO Nanofibrous Scaffolds

**Scheme 1:**
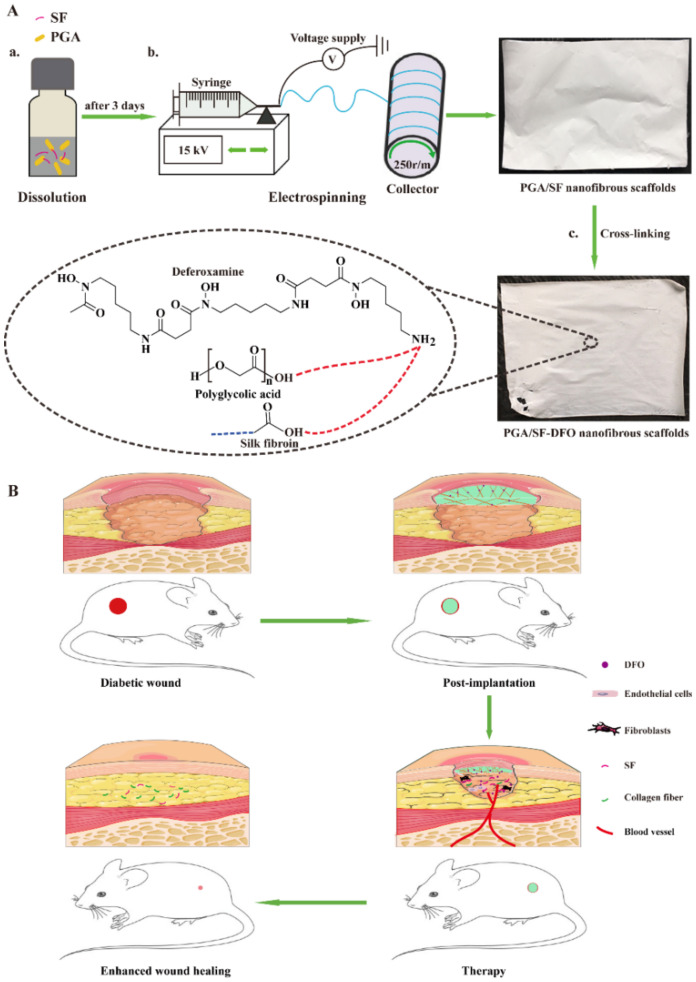
Schematic illustration of the preparation progress of PGA/SF-DFO nanofibrous scaffolds, the possible mechanism of wound healing activity of scaffolds, and their application for diabetic wound healing. (**A**) The preparation process of PGA/SF-DFO nanofibrous scaffolds. (**a**) Dissolution. (**b**) Electrospinning. (**c**) Cross-linking. (**B**) The possible mechanisms of wound healing activity of scaffolds and their application in diabetic wound healing.

Scheme 1 displayed the preparation process of PGA/SF-DFO nanofibrous scaffolds, the possible mechanism of wound healing activity of scaffolds, and their application in diabetic wound healing. Scheme 1A showed a three-step method to obtain PGA/SF-DFO nanofibrous scaffolds: Firstly, PGA and SF were dissolved in 5 mL HFIP and stirred for three days at room temperature. Next, PGA/SF nanofibrous scaffolds were obtained by electrospinning. Finally, DFO was incorporated into PGA/SF nanofibrous scaffolds to obtain PGA/SF-DFO scaffolds by the amido bond (CO-NH) formation under EDC/NHS solution for 24 h at room temperature. Scheme 1B presents the possible mechanisms of wound healing activity of scaffolds, and their application in diabetic wound healing. We supposed that SF free from the as-prepared scaffolds would achieve the desired results to remodel the mechanical properties of the diabetic wound as an ECM/collagen alternative and thus provide mechanical support for cells and guide cell behavior. Meanwhile, biodegradable nanofibrous scaffolds will make more space for new vessel formation and avoid secondary damage by omitting the process of scaffold removal. In addition, sustained release of DFO from scaffolds can induce angiogenesis and further enhance diabetic wound healing.

**Figure 1:**
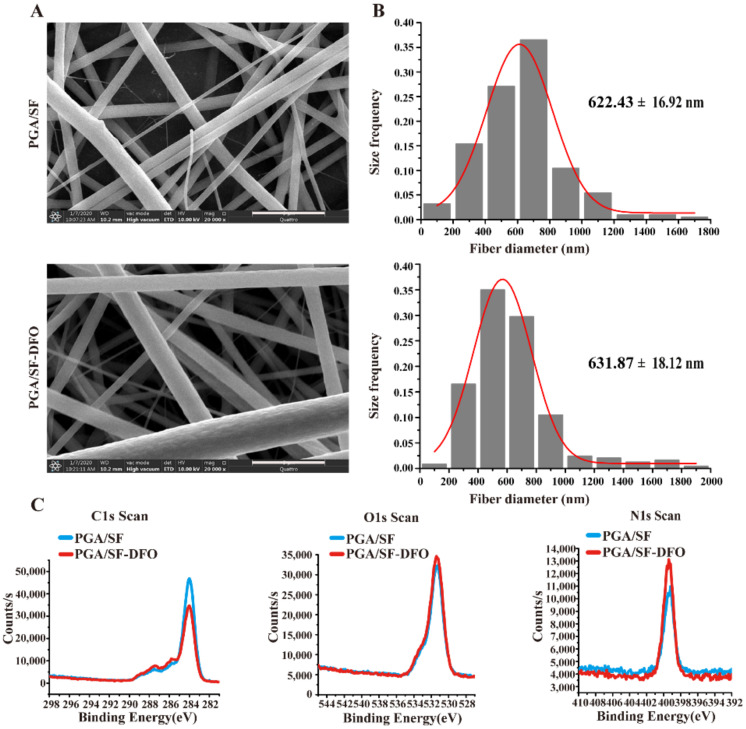
Characterization of PGA/SF and PGA/SF-DFO scaffolds. (**A**) SEM morphology. (**B**) The diameter distribution analysis of PGA/SF and PGA/SF-DFO scaffolds, scale bar = 5 μm. (**C**) The element analysis of C, N, O by XPS.

The results of SEM reveal the PGA/SF and PGA/SF-DFO scaffolds displayed relatively similar porous three-dimensional nanofibrous structures, which implies that PGA/SF scaffolds still retained the initial structure morphologies after the introduction of DFO (Figure 1A). Furthermore, this special structure mimics the natural extracellular matrix and further provides enough surface for adhesion and proliferation of cells, such as ECs, fibroblasts [54]. Besides, we found that the average diameters of PGA/SF-DFO nanofibers (631.87 ± 18.12 nm) showed a slight increase than that of PGA/SF nanofibers (622.43 ± 16.92 nm) (Figure 1B), which suggested that DFO may already adhere to the fibers. However, further analysis about the immobilization of DFO will be needed. In our study, we chose two methods, namely FTIR spectra, and XPS analysis, to confirm the immobilization of DFO into scaffolds. The results from FTIR spectra revealed the characteristic peaks of amide A (3302.14 cm^−1^), amide I (1653.58 cm^−1^), and amide II (1537.60 cm^−1^) present in SF [55] were observed at 3286.04, 1627.23, and 1523.6 cm^−1^ in the PGA/SF scaffolds, 3283.96, 1626.97, and 1524.24 cm^−1^ in the PGA/SF-DFO scaffolds, respectively. Besides, the characteristic peaks of PGA (carboxylic C=O band, 2957.75 cm^−1^) [56] appeared at 2958.85 cm^−1^ in the PGA/SF scaffolds to 2958.5 cm^−1^ in the PGA/SF-DFO scaffolds. In order to further estimate the immobilization of DFO into scaffolds, characteristic peaks of DFO [57] were also observed. Unfortunately, only the weak characteristic peak of DFO representing symmetric stretching vibrations (2864.13 cm^−1^) was observed, which implies that DFO was also incorporated on the PGA/SF scaffolds. However, others were not obvious. We speculate that this can be ascribed to two reasons: On the one hand, DFO belongs to small molecules and its incorporation does not affect the characteristic peaks of whole scaffolds. On the other hand, these characteristic peaks of DFO were next or overlapped to those of PGA or SF so that they cannot be distinguished from others. For example, the N-H stretching vibration (3325.57 cm^−1^) was close to that of SF observed at 3286.04 cm^−1^ in the PGA/SF scaffolds and 3283.96 cm^−1^ in the PGA/SF-DFO scaffolds, respectively. The characteristic peak of DFO, -CH2, representing asymmetric stretching vibration (2931.04 cm^−1^) was similar to that of PGA that appeared at 2958.85 cm^−1^ in the PGA/SF and 2958.5 cm^−1^ in the PGA/SF-DFO scaffolds, respectively (Figure S1). XPS analysis was completed to analyze the variation of elemental composition between PGA/SF and PGA/SF-DFO scaffolds. Figure 1C displays that the carbon element content in the PGA/SF-DFO scaffolds decreased and nitrogen element content increased when compared with that of PGA/SF scaffolds, which demonstrated that DFO was already incorporated in the PGA/SF scaffolds. Besides, we also found that oxygen element content was constant between the two groups. We speculate that the PGA/SF scaffold itself contains abundant oxygen element content so that the incorporation of DFO cannot change it. However, the content and distribution of immobilized DFO into the scaffolds need further study. The hydrophilicity or hydrophobicity of materials will be closely linked with their biocompatibility because hydrophilic scaffolds are more conducive to cell adhesion and growth [36,58]. The WCA analysis was completed to test the hydrophobicity of scaffolds. The data showed that the contact angles of all scaffolds were 0° (data not shown), which implies that our scaffolds are more hydrophilic. Abundant functional groups on the surface of PGA and SF, including –OH and –COOH, give them hydrophilicity [59,60]. Simultaneously, the good hydrophobicity indicates our scaffolds will have excellent biocompatibility and provide a moist environment to enhance wound healing.

### 3.2. Mechanical Properties of Different Scaffolds

**Figure 2:**
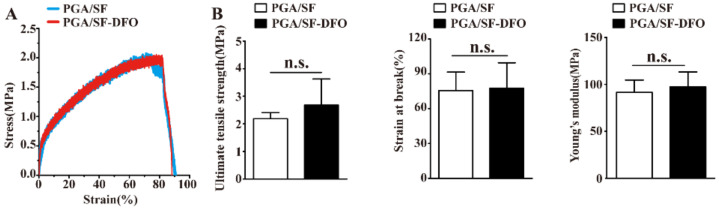
Mechanical properties of different scaffolds. (**A**) The stress–strain curves. (**B**) Ultimate tensile strength, strain at break, and Young’s modulus. n.s. represents no significant difference.

The mechanical property of diabetic skin is severely weakened, which is caused by abnormal extracellular matrix deposition, with particular attention to the lower level of collagen content [11,12,61]. Besides, scaffold material plays key roles in the field of tissue engineering, including supplying temporary and proper mechanical support and delivering physical and biochemical stimuli for cells [62,63,64]. Therefore, it is ideal for scaffold materials to have suitable mechanical properties. The results of the mechanical test showed that PGA/SF and PGA/SF-DFO scaffolds had similar stress–strain curves, confirming that the immobilization of DFO by cross-linking did not affect the scaffolds’ mechanical properties (Figure 2A). Besides, the ultimate tensile strength, strain at break, and Young’s modulus with 2.18 ± 0.22 MPa, 75.68 ± 15.91%, 91.58 ± 13.07 MPa, and 2.68 ± 0.94 MPa, 77.68 ± 21.85%, 97.34 ± 15.98 MPa, respectively, were no significant differences between the two groups (Figure 2B). The detailed data were shown in Table S1. However, we observed that the ultimate tensile strength of our scaffolds was relatively improved, compared with other scaffold materials [18,65], which may benefit from the excellent mechanical properties of PGA and SF [66,67]. 

### 3.3. Drug Release Behavior and Biodegradable Profile of Different Scaffolds

**Figure 3:**
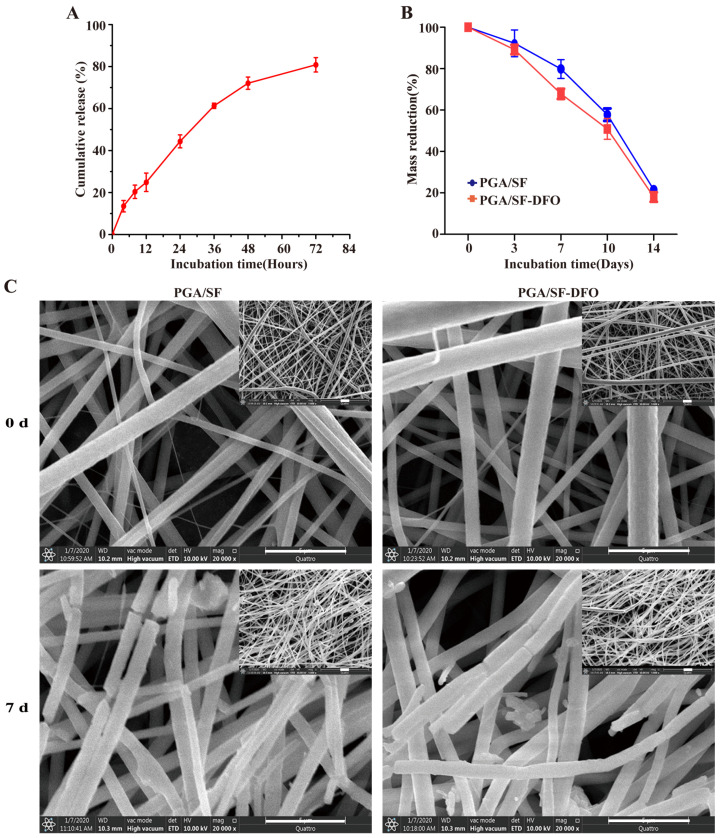
Drug release behavior and biodegradable profile of different scaffolds. (**A**) Cumulative release of DFO from PGA/SF-DFO scaffolds in vitro. (**B**) Mass reduction of different scaffolds over 14 days. (**C**) Representative SEM images of different degraded scaffolds at day 0 and 7 in vitro (scale bar = 5 μm).

The drug release profile in vitro was observed to investigate the release behavior of DFO from PGA/SF-DFO scaffolds. As shown in Figure 3A, we observed that the release profile of DFO experienced a burst release at the beginning. After that, the DFO release gradually showed a constant slow release, which inferred that the as-prepared scaffolds already achieved the sustained release of DFO for diabetic wound healing. At 72 h, the cumulative release of DFO reached 80.85%, which implied that abundant DFO was immobilized by cross-linking. This also hints us that physically absorbed DFO may preferentially release within the first few hours, and cross-linked DFO will occupy a large proportion at the later stage with scaffold degradation. Interestingly, we observed that the PBS incubated with scaffolds became muddy at 96 h in the process of DFO release, which reminds us that this phenomenon may attribute to scaffold degradation. Next, we also investigated biodegradable profiles of scaffolds in vitro. As shown in Figure 3B, the results demonstrated all the scaffolds degraded to almost 20% within 14 d, which inferred the application potential of our scaffolds to avoid secondary damage without removal of scaffold materials and make more space for re-vascularization with scaffold degradation. Meanwhile, the samples at 0 and 7 d were chosen to observe the variation of the surface structure of all the scaffolds by SEM. The micrographs intuitively showed us that all the scaffolds presented obvious cracks at 7 d in both two groups (Figure 3C). The results are consistent with the findings above.

### 3.4. Effects of PGA/SF and PGA/SF-DFO Scaffolds on Adhesion, Proliferation, Migration, and Tube Formation of HUVECs In Vitro

**Figure 4:**
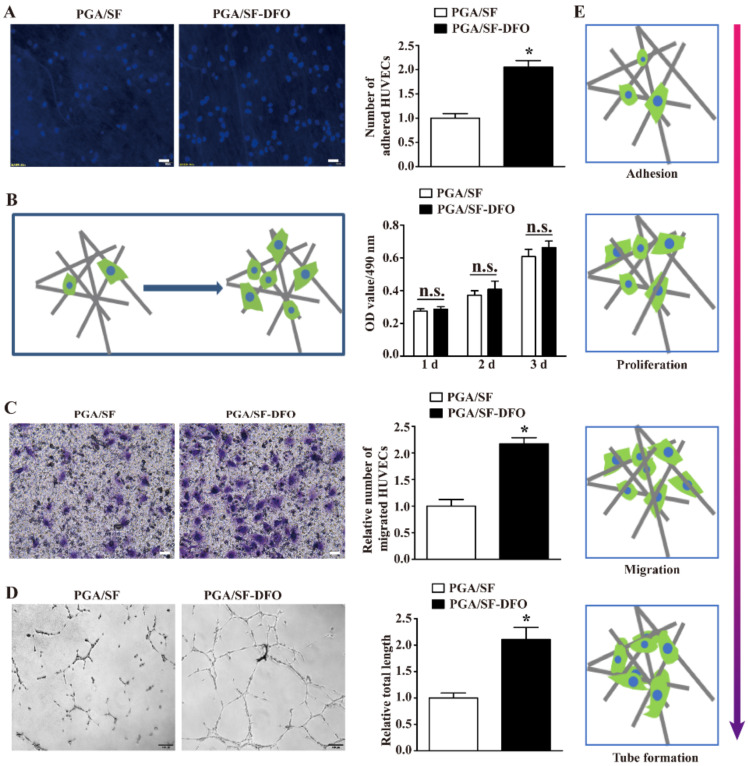
The effects of PGA/SF and PGA/SF-DFO scaffolds on HUVECs in vitro. (**A**) The representative images and quantitative analysis of HUVECs adhesion on the PGA/SF, PGA/SF-DFO scaffolds. Scale bar = 20 μm. (**B**) HUVECs proliferation. (**C**) HUVECs migration. Scale bar = 20 μm. (**D**) Tube formation of HUVECs. Scale bar = 100 μm. (**E**) Schematic illustration of adhesion, proliferation, migration, and tube formation of HUVECs in response to PGA/SF and PGA/SF-DFO scaffolds. Asterisks (*) indicate statistical significance, (*p* < 0.05), and n.s. represents no significant difference.

Considering the fact that all the scaffold materials must directly adhere to the wound to achieve a therapeutic effect, nice biocompatibility is necessary for ideal scaffold materials. In this study, HUVECs were seeded on different scaffolds to verify whether our scaffolds could meet the essential requirements. The adhesion ability of HUVECs on all the scaffolds was first observed. As shown in Figure 4A, these representative images and quantitative analysis indicated that more HUVECs adhered to PGA/SF-DFO scaffolds, when compared with that on the PGA/SF scaffolds, which suggests that PGA/SF-DFO scaffolds promote HUVECs adhesion. The cell proliferation of HUVECs on the scaffolds was continuously tested at 1, 2, 3 d by MTS assay. All the data demonstrated the cell proliferation of HUVECs was gradually increased in the same group in a time-dependent manner, but there is no statistically significant difference between PGA/SF and PGA/SF-DFO scaffolds (Figure 4B). These results indicated that DFO-loaded scaffolds had no significant toxicity for the viability and growth of HUVECs because of slow DFO release from the scaffolds, thus confirming our scaffolds already meet the requirement of acceptable biocompatibility for tissue engineering. A migration assay was performed in vitro to evaluate the influence of scaffolds on the migration ability of HUVECs by using a Transwell assay. The images and statistical data in this assay clearly demonstrated that PGA/SF-DFO scaffolds markedly stimulated the HUVECs migration compared to PGA/SF scaffolds (Figure 4C). Weak vascularization is the main characteristic of diabetic wounds [20,68] and seriously hampers the demand for oxygen, nutrients, and growth factors [33], which are necessary for wound healing, thus severely delaying wound healing [69]. Therefore, re-vascularization is pivotal to enhancing diabetic wound healing [18]. Herein, a tube formation assay was carried out to evaluate the angiogenic potential of the prepared scaffolds. After co-culture for 12 h, the PGA/SF-DFO scaffolds exhibited the best tube formation capacity of HUVECs when compared with the PGA/SF scaffolds (Figure 4D). The finding was due to the addition of angiogenic drug DFO because DFO has already been proved to activate HIF-1α and VEGF [70,71]. Figure 4E simply displayed the process of adhesion, proliferation, migration, and tube formation of HUVECs in response to the scaffolds, which play key roles in diabetic wound healing. Overall, the results for cell experiments in vitro confirmed our scaffolds were biocompatible with HUVECs regardless of the addition of DFO.

### 3.5. Scaffolds Degradation In Vivo 

**Figure 5:**
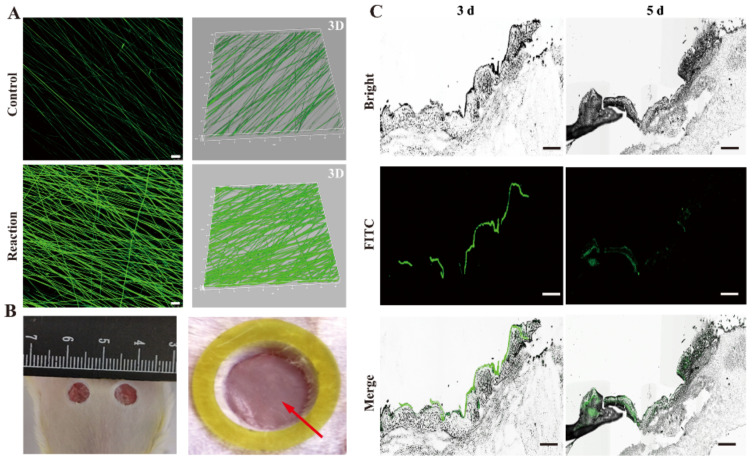
The degradation evaluation of scaffolds in vivo. (**A**) Representative confocal images (3D images at right side) of FITC labeled scaffolds. Control: physically absorbed FITC. Reaction: cross-linked FITC. Scale bar = 10 μm. (**B**) The implantation process of scaffolds. Red arrow: scaffold. (**C**) The biodegradability evaluation of scaffolds in vivo. Scale bar = 250 μm.

Although current wound dressings always display outstanding performance in diabetic wound healing, some disadvantages still limit their applications in clinical practice. For instance, an important issue in clinical practice is that it is difficult to remove wound dressings after treatments, which is easy to cause secondary damage, elicit pain, and even defer wound healing during dressing changes [72,73,74]. In order to address the issue, considerable efforts have been devoted toward fabricating effective, mild, and non-invasive changing dressings that would be employed in wound healing [75,76]. However, these efforts still cannot perfectly solve the issue. For example, dissolved dressings cannot be completely removed from the wound sites [72]. Therefore, biodegradable scaffold materials could be a better choice for wound healing. In our study, we chose two biodegradable raw materials of sutures, PGA and SF, to prepare scaffold materials for wound healing. In vitro, our scaffolds displayed excellent biodegradability. Here, we firstly prepared FTIC labeled scaffolds by two methods: physically absorbed and cross-linked to investigate the biodegradability of the PGA/SF scaffolds in vivo. The confocal and 3D images indicated that the density of FITC in the reaction group was obviously higher compared to control (Figure 5A), which demonstrated that scaffolds were successfully labeled by FITC under the EDC/NHS reaction system. Then, FTIC labeled scaffolds were implanted in the wound beds of diabetic mice (Figure 5B). Subsequently, we estimated the biodegradability of the PGA/SF scaffolds in vivo on days 3 and 5. The images on day 3 showed that the scaffolds (green color) were still comparatively integrated and adhered perfectly to the wound beds, indicating that our scaffolds have good adhesive capability and application potential for subsequent therapy as the therapeutic effect is insufficient when the scaffold materials are out of the wound. Intuitively, the as-prepared scaffolds have already been degraded to a certain extent at day 5 (Figure 5C), which demonstrated the potential of scaffolds used for diabetic wound healing to avoid secondary damage by omitting the removal process of scaffolds. Meanwhile, the finding showed us that SF free from as-prepared scaffolds with PGA degradation might grow into the wound to remodel the mechanical properties of diabetic wound and thus provide mechanical support for cell adhesion, growth, and migration as a temporary and transitional ECM/collagen alternative.

### 3.6. In Vivo Wound Healing

**Figure 6:**
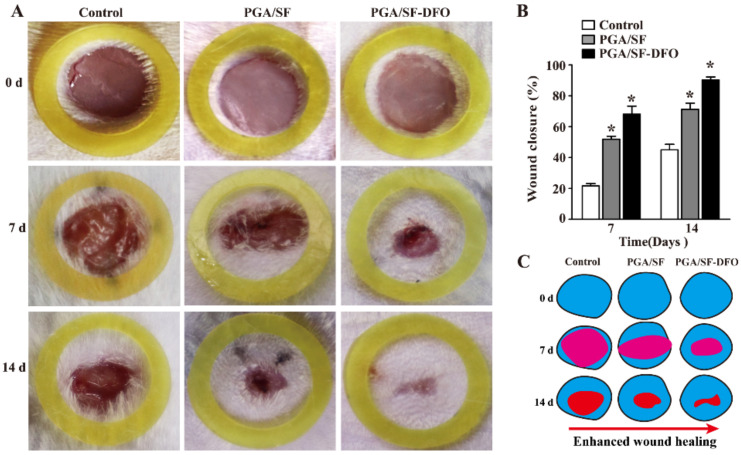
Diabetic wound healing evaluation of PGA/SF-DFO scaffolds. (**A**) Digital photographs of the wounds. (**B**) Quantification of the wound closure by Image J software. (**C**) Schematic diagram of wound healing at days 0, 7, and 14 after injury. Asterisks (*) indicate statistical significance, (*p* < 0.05).

To further verify the potential of scaffolds in diabetic wound healing, the repair efficacy of scaffolds in vivo was observed and summarized at days 0, 7, and 14. As shown in Figure 6A, the images displayed the fastest wound repair were observed in the PGA/SF-DFO group among all the groups. In addition, the speed is faster in the PGA/SF scaffold group compared to the control. Wound closure (%) and schematic diagram in each group were displayed in Figure 6B,C. It can be seen that the newly formed skin had almost covered the 90.37% of wound surface at 14 d in the PGA/SF-DFO group while it is 71.32% and 54.17% in the PGA/SF and control group, respectively. The high wound closure rate may attribute to several key characteristics of PGA/SF-DFO scaffolds in comparison with those of similar silk fibroin-based composites reported previously (Table S2) [3,77,78,79,80]. In addition, this finding indicated pure PGA/SF scaffolds have certain positive wound repair effects with scaffold degradation and the addition of DFO could further magnify the wound repair effects of the PGA/SF scaffolds as its proangiogenic activity.

### 3.7. Silk Fibroin-Based Scaffolds Remodeled the Mechanical Properties of Wounds

**Figure 7:**
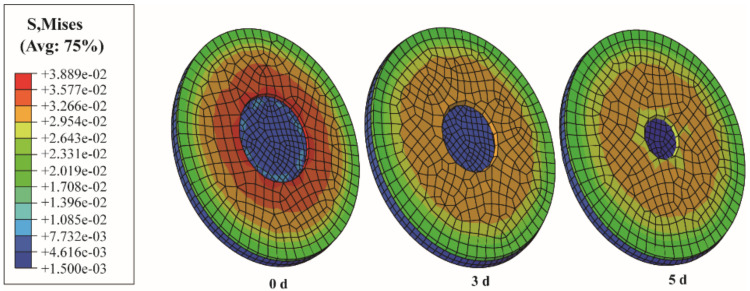
The finite element model showing distribution of local Mises stress in wound and surrounding tissue.

In order to further investigate the effect of silk fibroin-based scaffolds on the mechanical properties of wounds, we used finite element modeling to explore local changes in mechanical stress and strain in the wounds. Using previous research data on mechanical loading of skin tissue and local stiffness of wound and its surrounding skin as inputs, the finite element modeling quantified the local stress or strain in and around the wound. As silk fibroin-based scaffolds degraded, the subcutaneous layer accumulates and gradually thickens with diabetic wound healing. Therefore, we simulated the stress distrib ution in and around the wound at special time intervals. As shown in Figure 7, the results of finite element analysis showed that there was stress concentration (red section) at day 0 with maximum stress of 3.889 × 10^−2^ MPa around the wound after operation. Nevertheless, the application of silk fibroin-based scaffolds could significantly reduce the maximum stress around the wound at days 3 and 5 with maximum stress of 3.468 × 10^−2^ MPa and 3.331 × 10^−2^ MPa, respectively. This finding suggested that silk fibroin-based scaffolds indeed remodeled the mechanical properties of wounds, which would provide favorable mechanical microenvironment for cells and thus enhance wound healing.

### 3.8. Histopathological Analysis 

**Figure 8:**
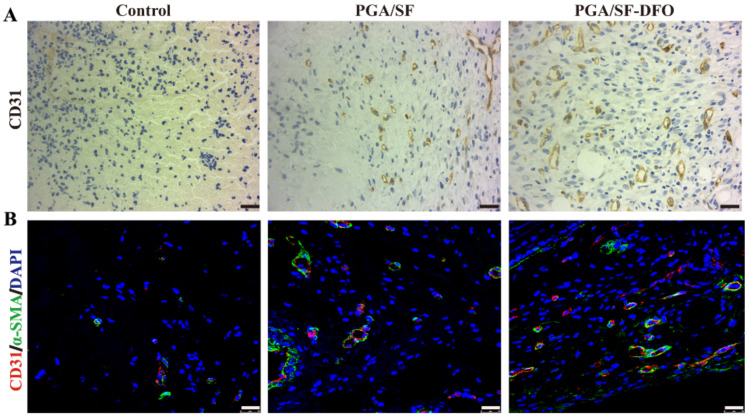
IHC and IF analysis of re-vascularization in wound beds at day 7 post-implantation. (**A**) IHC staining analysis with CD31 and (**B**) IF analysis with CD31 and α-SMA. Scale bar = 25 μm.

Angiogenesis, involved in the formation of new blood vessels, plays a key role in diabetic wound healing. Therefore, it is essential to induce angiogenesis and further enhance diabetic wound healing for our scaffolds. To further examine the effect of PGA/SF-DFO scaffolds on the formation of blood vessels in vivo, immunohistochemical (IHC) for CD31 and double immunofluorescence (IF) staining for CD31 (red) + α-SMA (green) were conducted. The images of CD31 obtained by IHC staining confirmed that PGA/SF-DFO scaffolds induced the highest revascularization in the wound at day 7 post-implantation compared to other groups. In addition, higher revascularization was observed in the PGA/SF group compared to that in the control group (Figure 8A). A similar result was obtained by double immunofluorescence staining (Figure 8B). These encouraging outcomes were consistent with previous studies [70,81], thus confirming the application potential of our scaffolds in clinical practice. 

**Figure 9:**
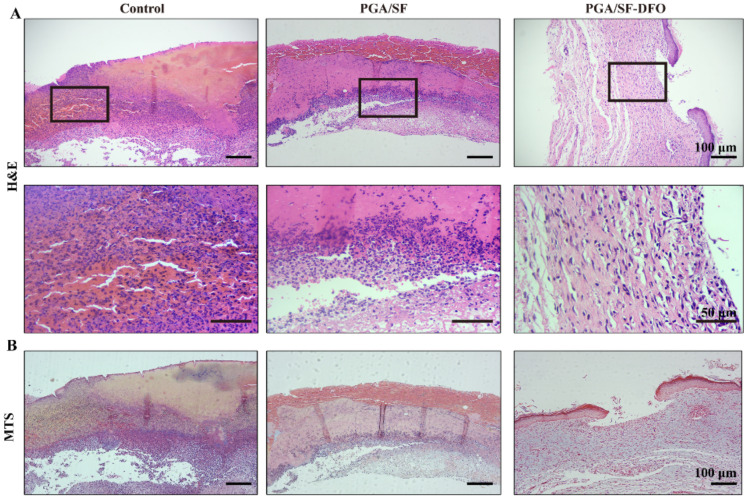
Histopathological analysis of the wound beds at day 7 post-implantation. (**A**) H&E staining of wound beds. (**B**) Masson’s trichrome staining of wound beds.

Wound healing is a process containing several well-organized and overlapped stages: hemostasis, inflammation, cell proliferation, and remodeling [47]. A histological analysis was conducted to further investigate the wound repair effect of as-prepared scaffolds. The data of H&E staining was shown in Figure 9A. It can be seen that the residual scabs, such as crusts of dried blood, exudate, and serum, were still observed in both PGA/SF and control groups, confirming that the diabetic wound healing is delayed in these two groups. In addition, the wound surface crusts in both PGA/SF and control groups were infiltrated by acute inflammatory cells, which would further delay the healing process of diabetic wound healing. Comparatively, fewer residual scabs or inflammatory cells were found in the PGA/SF group than that in the control group, which demonstrated that pure PGA/SF scaffolds were still effective for wound healing to a certain extent. As expected, there are no residual scabs observed in the PGA/SF-DFO scaffold group. Meanwhile, the PGA/SF-DFO scaffold group displayed a mild inflammatory microenvironment, which is favorable for diabetic wound healing. As the raw materials of new skin tissue, collagen deposition was thought to be an intuitive evaluation indicator of wound repair [82]. The images of Masson’s trichrome staining showed that PGA/SF-DFO scaffolds obviously accelerated the collagen deposition in the wound when compared with PGA/SF and control groups (Figure 9B). This indicated that PGA/SF-DFO scaffolds exhibited a greater positive effect to induce collagen deposition, thus confirming the potent therapeutic performance of our scaffolds.

### 3.9. Evaluation of Mechanical Properties of Impaired Wounds with Different Scaffolds

**Figure 10:**
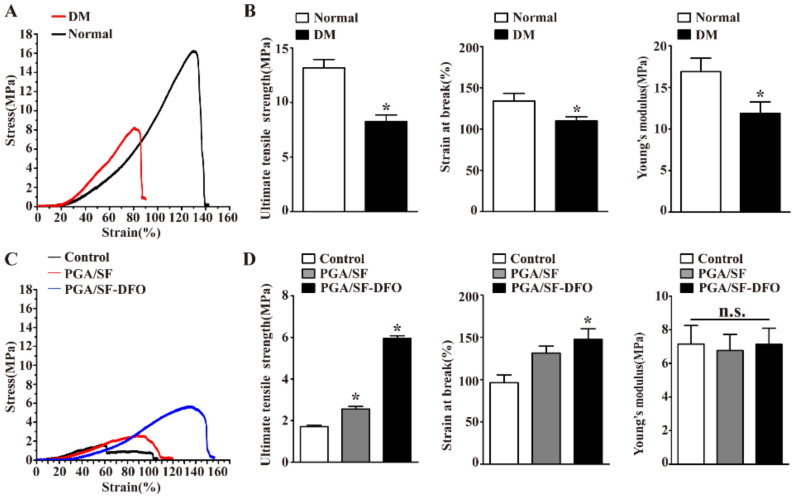
Mechanical properties of the skin/wound beds. (**A**) The stress–strain curves of skin from normal and diabetic mice (DM). (**B**) Ultimate tensile strength, strain at break, and Young’s modulus of skin from normal and diabetic mice (DM). Asterisks (*) indicate statistical significance, (*p* < 0.05). (**C**) The stress–strain curves of wound bed from control, PGA/SF, PGA/SF-DFO group at day 14 post-implantation. (**D**) Ultimate tensile strength, strain at break, and Young’s modulus of skin from control, PGA/SF, PGA/SF-DFO group at day 14 post-implantation. Asterisks (*) indicate statistical significance, (*p* < 0.05), and n.s. represents no significant difference.

The mechanical properties of skin play an important role in various applications [83]. However, Bermudez et al. reported that mechanical properties of diabetic skin were impaired in comparison to that of nondiabetic skin [84] as diabetes altered the structure and composition of ECM, especially collagen, and subsequently mechanical function. Here, we chose an electromechanical dynamic/static testing system (Instron E1000, High Wycombe, UK) to verify the results in our diabetic model. The data presented in Figure 10A,B showed that the stress, strain at break, and Young’s modulus of skin from diabetic mice (8.25 ± 0.61 MPa, 109.84 ± 5.13%,11.92 ± 1.34 MPa) were significantly lower when compared to that from normal mice (13.17 ± 0.76 MPa, 134.05 ± 9.34%, 16.91 ± 1.62 MPa), which indicated that diabetes seriously impaired the mechanical properties. The finding is concordant with the previous study [84]. The detailed data were summarized in Table S3. Our scaffolds have already been proved to improve angiogenesis and collagen deposition of the diabetic wound, which play key roles in maintaining the mechanical properties of the skin [83]. Thus, the recovery of mechanical properties of the wound beds is thought to be a direct indicator of functional recovery of skin wound. We investigated the mechanical properties of wound beds at 14 d post-implantation from the control, PGA/SF, and PGA/SF-DFO groups by the method described before. As shown in Figure 10C,D, the data showed that the mechanical strength of wound beds was significantly improved by PGA/SF and PGA/SF-DFO scaffolds with the ultimate tensile strength of 2.55 ± 0.13 MPa and 5.95 ± 0.13 MPa, respectively, when compared with the untreated control group (1.71 ± 0.07 MPa). As compared with previously unwounded diabetic skin (8.25 ± 0.61 MPa), PGA/SF and PGA/SF-DFO scaffolds improved the mechanical strength of impaired wound up to about 30%, 70%, respectively. The detailed information including ultimate tensile strength, strain at break (%), and Young’s modulus (MPa) is displayed in Table S4. Taken together, these encouraging data we obtained indicate that a combination of PGA, SF, and DFO in the PGA/SF-DFO scaffolds could more efficiently accelerate diabetic wound healing.

## 4. Conclusions

In the present study, we successfully prepared mechanic-driven biodegradable PGA/SF-DFO scaffolds by the introduction of two raw materials of sutures, PGA, SF, and an angiogenic agent DFO using electrospinning technique. The experiments in vitro demonstrated multiple physicochemical characteristics of as-prepared scaffolds, including porous three-dimensional structures with high surface area, favorable mechanical properties, hydrophilicity, and high biocompatibility. Moreover, PGA/SF-DFO scaffolds displayed moderate biodegradability in vitro and in vivo, which would provide the application potential to avoid secondary damage by omitting the removal process of scaffold materials and make more space for re-vascularization with scaffold degradation. Besides, the finite element analysis results showed that the application of silk fibroin-based scaffolds could significantly reduce the maximum stress around the wound. PGA/SF-DFO scaffolds could enhance diabetic wound healing by promoting vascularization, collagen deposition, and recovery of the mechanical properties of the wound beds. In summary, PGA/SF-DFO scaffolds combined the advantages of PGA, SF, and DFO and further enhanced diabetic wound healing, providing a promising therapeutic strategy for clinically diabetic wound healing.

## Data Availability

The data presented in this study are available on request from the corresponding author.

## Supplementary Materials

The following supporting information can be downloaded at: https://www.mdpi.com/article/10.3390/pharmaceutics14030601/s1, Figure S1: FTIR spectra for SF, PGA/SF, PGA/SF-DFO, PGA, DFO. Red arrow: the weak characteristic peak of DFO representing symmetric stretching vibrations at 2864.13 cm^−1^; Table S1: Ultimate Tensile Strength, Strain at Break and Young’s Modulus of Scaffolds; Table S2: Summary of several key characteristics of PGA/SF-DFO scaffolds in comparison with other silk fibroin-based composites. Table S3: Ultimate Tensile Strength, Strain at Break and Young’s Modulus of Skin from Normal and Diabetic Mice; Table S4: Ultimate Tensile Strength, Strain at Break and Young’s Modulus of Wound Bed from Control, PGA/SF, PGA/SF-DFO Group at Day 14.
