# Supplementary material for: Mechanic-Driven Biodegradable Polyglycolic Acid/Silk Fibroin Nanofibrous Scaffolds Containing Deferoxamine Accelerate Diabetic Wound Healing
Source: Pharmaceutics. 2022 Mar 10;14(3):601. doi: 10.3390/pharmaceutics14030601 (PMC8948832; doi:10.3390/pharmaceutics14030601)
Supplement: Supplementary file 1 [file pharmaceutics-14-00601-s001.zip › pharmaceutics-1596305-supplementary.pdf]

pharmaceutics

Mechanic-Driven Biodegradable Polyglycolic Acid/Silk Fibroin Nanofibrous Scaffolds Containing Deferoxamine Accelerate Diabetic Wound Healing

Shenfang Zha, Yohanes Kristo Sugiarto Utomo, Li Yang, Guizhao Liang* and Wanqian Liu*

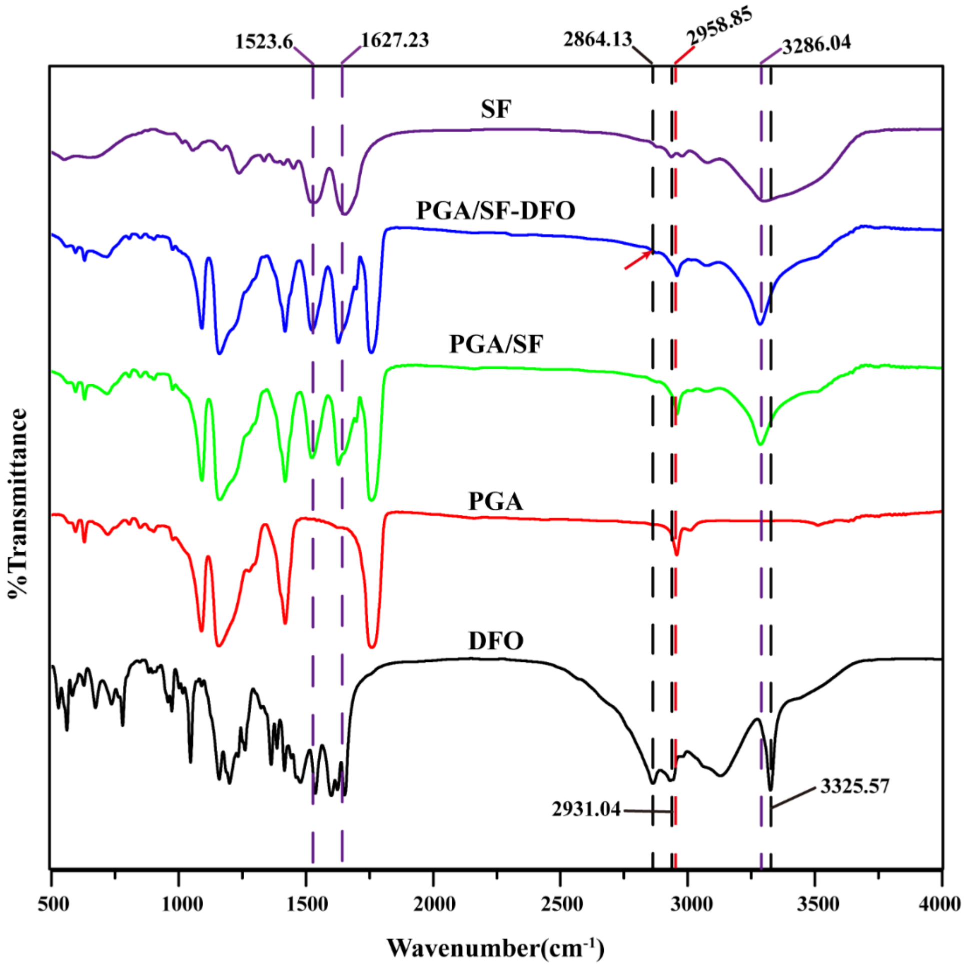

Figure S1. FTIR spectra for SF, PGA/SF, PGA/SF-DFO, PGA, DFO. Red arrow: the weak characteristic peak of DFO representing symmetric stretching vibrations at 2864.13 cm^{-1}.

Table S1. Ultimate Tensile Strength, Strain at Break and Young's Modulus of Scaffolds.

	ultimate tensile strength (MPa)	strain at break (%)	Young's modulus (MPa)
PGA/SF	2.18 ± 0.22	75.68 ± 15.91	91.58 ± 13.07
PGA/SF-DFO	2.68 ± 0.94	77.68 ± 21.85	97.34 ± 15.98

Table S2. Summary of several key characteristics of PGA/SF-DFO scaffolds in comparison with other silk fibroin-based composites.

Composite	Drug	Sustained release time	Tensile strength (MPa)	Mass reduction(%)	Would closure rate	Targeted application	References
PGA/SF-DFO	DFO	80% at 72 h	2.68 ± 0.94	Degradation of 80% at 14 d	90.37% at 14 d	Diabetic wound healing	This work
SF/CS/SA with DS	DS	50% at 100 min	2.63± 0.35	-	-	-	77
CU-SF-PCL	CU	53.48 ± 5.21% at 12 h	17.54± 1.3	Degradation of 90% at 14 d	91.67% at 14 d	Diabetic wound healing	3
CU-SF-PVA	CU	75.48 ± 3.85% at 12 h	12.41 ± 1.24	Completely degradation on day 4	94.95% at 14 d	Diabetic wound healing	3
AS-loaded SF/GT	AS	80% within 12 h and over 95% at 36 h	-	-	Over 90% at 14 d	Wound healing	78
SF-fenugreek (1:1)	Fenugreek	73 ± 0.9 % within 24 h	4.57 ± 0.19	-	95.8 % at 12 d	Excisional wound healing	79
HA–SF–3ZO	ZO	over the period of 14 days	7.12 ± 0.17	Degradation of 24.65 ± 1.76% during 1 week	55.02 ± 1.35% at 7 d	Burn wound healing	80

CS-Chitosan; SA-Sodium alginate; DS-diclofenac sodium; CU-Curcumin; PVA-polyvinyl alcohol; PCL-polycaprolactone; AS-astragaloside; GT-gelatin; HA-hyaluronic acid; ZO-zinc oxide.

Table S3. Ultimate Tensile Strength, Strain at Break and Young's Modulus of Skin from Normal and Diabetic Mice.

	ultimate tensile strength (MPa)	strain at break (%)	Young's modulus (MPa)
Normal skin	13.17 ± 0.76	134.05 ± 9.34	16.91 ± 1.62
Diabetic skin	8.25 ± 0.61	109.84 ± 5.13	11.92 ± 1.34

Table S4. Ultimate Tensile Strength, Strain at Break and Young's Modulus of Wound Beds from Control, PGA/SF, PGA/SF-DFO Group at Day 14.

	ultimate tensile strength (MPa)	strain at break (%)	Young's modulus (MPa)
control	1.71 ± 0.07	96.39 ± 9.18	7.16 ± 1.09
PGA/SF	2.55 ± 0.13	131.40 ± 8.11	6.77 ± 0.96
PGA/SF-DFO	5.95 ± 0.13	147.58 ± 12.50	7.13 ± 0.97
